# Annotations

**Published:** 1910-01-08

**Authors:** 


### Annotations.

### Medical Council for Canada.

At a special meeting held recently by the Ontario Council of the College of Physicians and Surgeons, it was proposed to create a Medical Council for Canada. The proposed Council will have the co-operation and support of the provincial bodies, and holders of its qualification will have the power to practise medicine in all the provinces. A register, boards of examiners, and a status of the profession, insuring recognition in Great Britain, are provided for, and the powers of the Council are defined. It is to be composed of three members from all the provinces, appointed by the President in Council; a number of members representing each province in proportion to the number of practitioners, one member from each University or incorporated medical college, and one member from each district school of medicine. The Council will elect officers from its members, and appoint a registrar and other officers deemed necessary. If this proposal becomes law, provincial medical reciprocity will be virtually established. The medical profession of Quebec will probably, however, block the passage of such a Bill, as the majority of its members appear to be opposed to [illegible]ter-provincial reciprocity.

### The Importance of Play.

Dr. T. S. Clouston has a deservedly high reputation throughout Scotland for knowledge, ability, and common sense. He spent the best years of his life as physician superintendent of the Royal Edinburgh Asylum for the Insane, which he developed immensely, and where he brought the treatment of mental cases to a high standard of efficiency. Lecturing before the Child Study Society recently, he laid it down that, in the case of children, whilst we have little control over heredity, we should possess absolute control over environment. The effects of environment are such that they may make or mar the mental development of a child, may equip it physically to face the world and its work with ease, or leave it a relatively miserable, anæmic incapable. These environments include the outward forces of nature, such as light and air. At birth the child possesses no mind at all, and if it is deprived of sight and hearing it will remain in a condition allied to idiotcy. Such a child, brought up in darkness, would be mutilated in mind, and would not develop thought or feeling or conduct. To state this is to condemn every form of local government which suffers insanitary houses and overcrowded dwellings to remain within the area of its jurisdiction. The members of such bodies who permit these evils will one day have a heavy account to settle for their misdeeds through the neglect of a plain public duty. We wish that a prophet of the old type might arise to drive this fact home to every city and county council and to every local authority, because then we believe it would soon be impossible to find anywhere types of the abominable dwellings which are still occupied by our poorer countrymen up and down the country. Town life under the best conditions is apt to make children unstable in mind. Yet stability of mind is the most desirable quality for any growing human being. It follows that the children of all residents in towns should be secured good food, plenty of fresh air and sunlight, and the fullest opportunities for play and exercise. No city, or town, or rural community for that matter, is entitled to regard itself as modern which does not possess adequate playgrounds for its children. Who can estimate the loss to a nation which arises from the absence of playgrounds, whereby the physical and moral characters of men and women are materially affected for evil everywhere?

### Sex in Medicine.

At the commencement of a new year we may properly advocate more manliness in medicine and the exhibition of greater self-respect where competition is keenest. The medical profession is suffering increasingly from the advocates of enforced protection for its members, which is tending to discredit the noblest of all callings and to degrade it to the level of a trade or business. This state of things is bad enough, but it would soon become worse if the attitude attributed to certain members of the profession in Preston, as reported in the papers, were to be generally followed in regard to the appointment of qualified women as medical officers of girls' schools and hospitals. Alderman Hamilton, speaking at a meeting of the Town Council of Preston, declared that the medical profession were making a determined effort to prevent the introduction of a lady doctor into the town. The trouble has arisen over the appointment of a qualified medical inspector to Park School. The Board of Education recommended a lady doctor, and the head mistress had made representations to the same effect. These recommendations were strongly opposed by the medical men of Preston, according to Alderman Hamilton, on the ground "that the introduction of a lady doctor would interfere with their practice, that she would get access to some of their patients, and might ultimately divert them from their regular medical practitioners." This statement, which is *ex parte*, will, we hope, be contradicted by the profession in Preston, for it is dishonouring as it stands. There is plenty of room for women, as well as for men, who qualify as medical practitioners. Any man, or for that matter any woman, who loses a patient to the other sex probably owes the loss to relative incompetence, and to no other cause, for sex can have little or nothing to do with the matter, seeing that, once qualified, a practitioner of either sex is bound by the same regulations and the same code of honour. The increase in the number of qualified women ought, therefore, to put men on their mettle and ensure that the weaker brethren, by post-graduate and other methods, keep themselves well up to date, so that they may retain their patients against all comers. We shall be glad to hear from the profession in Preston, but, meantime, on behalf of the whole profession, we must emphatically repudiate the implication made against it in the speech of Alderman Hamilton, from which we have quoted.

